# Effects of a Carbohydrate Meal on Lipolysis

**DOI:** 10.3390/nu16203531

**Published:** 2024-10-18

**Authors:** Kerstin Kempf, Stephan Martin

**Affiliations:** 1 West-German Centre of Diabetes and Health, Düsseldorf Catholic Hospital Group, 40591 Dusseldorf, Germany; stephan.martin@uni-duesseldorf.de; 2 Medical Faculty and University Hospital Dusseldorf, Heinrich-Heine-University Dusseldorf, 40225 Dusseldorf, Germany

**Keywords:** insulin, ketosis, ketone, ketogenic diet, acetone, lipolysis, carbohydrates, overweight, obesity, type 2 diabetes, weight loss

## Abstract

Background: Due to the increasing prevalence of obesity and type 2 diabetes, effective dietary recommendations are needed. Previously, we developed the low-insulin method: by avoiding insulinogenic, i.e., insulin-release-triggering foods, insulin secretion becomes reduced, lipolysis is stimulated, and energy production is shifted to ketosis with excess ketone bodies exhaled in the form of acetone. Now, we investigate how quickly stable ketosis (defined as fasting breath acetone concentration ≥ 7.0 ppm) is achieved, whether and for how long a carbohydrate meal inhibits ketosis, and whether the responses differ in healthy adults with different insulin levels. Methods: An oral glucose tolerance test was conducted, and body composition and fasting insulin were determined at the beginning and end of the 14-day study. Participants (*n* = 10) followed a ketogenic diet and performed continuous glucose monitoring. Ketosis levels were determined by measuring breath acetone concentrations. On day 8, two white bread rolls with jam (72 g carbohydrates) were consumed for breakfast. Results: After seven days, all participants achieved stable ketosis (defined as fasting breath acetone concentration ≥ 7.0 ppm), which dropped from 8.2 to 5.7 ppm (*p* = 0.0014) after the carbohydrate meal. It took five days to achieve stable ketosis again. The stratification of participants into tertiles according to their fasting insulin levels demonstrated that individuals with low fasting insulin levels achieved stable ketosis again after two days and those with medium insulin levels after five days, while those with high baseline values did not reach stable ketosis by the end of the study. Conclusions: By carbohydrate restriction, stable ketosis can be achieved within one week. However, a single carbohydrate meal inhibits ketosis for several days. This effect is pronounced in individuals with elevated fasting insulin levels.

## 1. Introduction

The multitude of dietetic recommendations is confusing for people who want to lose weight [1]. In addition, lifestyle intervention studies have unanimously shown that weight increases again as soon as intensive accompaniment ends or the participants fall back into old behaviour patterns [2,3]. Due to the increasing prevalence of overweight, obesity [4] and overweight-associated illnesses such as type 2 diabetes mellitus, it is absolutely necessary to understand the mechanisms of weight loss and weight gain in order to provide dietary recommendations that enable successful and long-term weight reduction.

In the past, nutritional advice was mainly focused on reducing dietary fats rather than carbohydrates [5]. Long-term studies demonstrated the beneficial and protective effects of nutritional fats. The elevated use of fats was associated with a lower risk of weight gain [6], type 2 diabetes [7], cardiometabolic diseases, or all-cause mortality [8], especially in contrast to carbohydrates [9]. Thus, a low-carb diet demonstrated superiority in the context of weight loss and metabolic improvements compared to a low-fat diet [10]. It has long been known that every carbohydrate-containing meal provokes an insulin secretion, which, in addition to its primary function of glucose uptake into target cells, leads to the effective inhibition of lipolysis [11,12,13,14]. The insulin concentrations that lead to the inhibition of lipolysis are 10–100 times lower than the concentrations required for the acceleration of glucose metabolism [15].

Earlier studies have shown that in this context the metabolic state plays an essential role. While people with a beneficial metabolic state tolerate glucose ingestion well, it has been shown that people with lacking metabolic flexibility (e.g., through an increased body mass index (BMI), impaired glucose tolerance, etc.) become impaired [16,17,18]. In obese people, insulin levels are not only elevated in a fasting state, but they also increase much more after glucose consumption and remain high longer than in people with normal weight [18]. This led us to the hypothesis that in people with metabolic impairment (due to hyperinsulinaemia, obesity, type 2 diabetes, etc.), carbohydrate consumption provokes a more intense inhibition of lipolysis, rendering weight loss much more difficult.

Our research focuses on the negative effects of insulin on weight development and their prevention. In previous work, we were able to show that hyperinsulinaemia is negatively associated with weight loss [19] and that the reduction in elevated fasting insulin levels is the basis for successful weight reduction [20]. In this context, we tested different kinds of food for their insulinogenic properties [21]. Thus, changing a standard carbohydrate-containing bread for a low-insulin bread led to a significant weight loss in study participants who had not changed their lifestyle otherwise [22]. Based on these results, we developed the low-insulin method [23], a lifestyle intervention focusing on the avoidance of insulinogenic foods and behaviour. Thus, insulin secretion becomes reduced, lipolysis is stimulated, and energy production is shifted to ketosis.

Ketone bodies, i.e., acetone, acetoacetate, and β-hydroxybutyrate, are produced in liver cells by the breaking down of fatty acids. Ketosis is considered as any state in which the liver produces ketone bodies more rapidly than they can be depleted by the tissues [24]. They are released into the blood after liver glycogen stores have been exhausted, which typically occurs within the first 24 h of fasting. In stable ketosis, ketone bodies are used for energy generation, while excess ketone bodies are eliminated via urine or breathing air [25]. Since a strong correlation exists between breath acetone concentration and the rate of fat loss [26], we tested a recently available technological approach, i.e., measuring breath acetone concentration as a marker for ketosis levels and lipolysis in healthy volunteers.

In order to obtain further insight into the mechanistic effects of lipolysis, the present study investigated (i) how quickly stable ketosis can be achieved in healthy adults, (ii) whether and for how long a high-carbohydrate meal inhibits ketosis, and (iii) whether the responses differ in individuals with different fasting insulin levels.

## 2. Materials and Methods

### 2.1. Study Design and Study Subjects

This single-group non-blinded intervention study was conducted at the West German Centre of Diabetes and Health, Düsseldorf, Germany. A flyer at the study centre informed people about the possibility of study participation. Eligible participants were healthy adults, 18–65 years old with a BMI of 18–35 kg/m^2^. Exclusion criteria were acute diseases, diabetes mellitus (fasting glucose ≥ 126 mg/dL or diabetes-related medication), severe illness with in-patient treatment during the last 3 months, pregnancy or breast-feeding, medication for weight reduction, and weight change > 2 kg/week during the last month.

### 2.2. Intervention

The participants followed a ketogenic diet for 14 days with less than 50 g digestible carbohydrates per day. On day 8, they consumed a carbohydrate-containing breakfast consisting of two white bread rolls with jam (=72 g of digestible carbohydrates).

### 2.3. Measurements

On the first day and on day 14, participants visited the study centre after an overnight fast of at least 10 h for the determination of anthropometric and clinical data (age, sex, body weight, height, BMI, hip and waist circumference, blood pressure, lean and fat mass, and fasting insulin). Body weight was measured in light clothing to the closest 0.1 kg, height to the closest 0.5 cm. Waist circumference was measured at the minimum abdominal girth about midway between the rib cage and the iliac crest, and hip circumference was measured at the largest circumference around the buttocks. Body composition was measured using a state-of the-art body composition scale (Seca mBCA515, Seca, Hamburg, Germany). Blood pressure was determined on both arms in a sitting position after a 5 min rest.

Before the start of the intervention, a standardised 75 g oral glucose tolerance test (OGTT) (DextroOGT; Roche, Mannheim, Germany) was performed. For this purpose, an intravenous cannula was inserted into the forearm vein and 13 mL blood was taken before as well as 1 and 2 h after oral glucose load. At the local laboratory, blood glucose was measured by photometry with an intra-assay coefficient of variability (CV) of 1.9%, and plasma insulin by electrochemoluminescence immunoassay (ECLIA) with a CV of 3.6%.

During the 14 days of intervention, glucose levels were measured using continuous glucose monitoring (FreeStyle Libre 3; Abbott Diabetes Care, Alameda, CA, USA). In detail, on the first day the glucose sensor was attached to the upper arm. Glucose recordings were automatically performed every 15 min over a period of 14 days. Data were downloaded by scans with a handheld device. According to the ISO 15197:2013 [27], the accuracy of the sensors had been determined to be 73.2% (i.e., the percentage of values within ±15 mg/dL of the reference value at glucose concentrations < 100 mg/dL and within ±15 % at glucose concentrations ≥ 100 mg/dL [28]).

Additionally, participants were trained on the first day to determine their ketosis level as a measurement for lipolysis rate by measuring breath acetone concentration at eight defined time points. Breath samples were measured by blowing the exhaled breath after a 2 s breath hold into a handheld device (KetoScan mini, KetoScan, Freilassing, Germany). The device additionally requests a breath sample, which is used as a reference during the diagnosis. Participants were instructed not to consume alcohol during the study, not to drink beverages other than water, i.e., coffee, juice, etc., and not to eat, smoke or brush their teeth (including using mouthwash) within 30 min before the measurement.

### 2.4. Outcomes

The primary outcome was the difference between the fasting breath acetone concentration before and after carbohydrate consumption. Secondary endpoints were the time for reaching a significant ketosis level in the complete cohort as well as stratified by fasting insulin levels at baseline.

### 2.5. Statistics

The sample size calculation was performed using G*Power 3.1.9.2., estimating a difference of 2.5 ppm in fasting acetone exhalation due to carbohydrate consumption. In order to identify such a difference with a power of 95%, a level of significance of 5%, and an estimated dropout rate of 10%, 10 persons had to be recruited. Intention-to-treat (ITT) analyses were performed and all available data, including outliers, were included in the analysis. Normality was analytically confirmed, applying the D’Agostino and Pearson omnibus normality test. Non-normally distributed data, i.e., differences between day 1 and day 14, were analysed by the Wilcoxon signed rank test. For normally distributed data, i.e., breath acetone concentration on day 8 vs. day 9, paired *t*-tests were used. All statistical tests were two-sided, and the level of significance was set at *p* = 0.05. All analyses were performed using GraphPad Prism 6.04 (GraphPad Software, San Diego, CA, USA).

## 3. Results

### 3.1. Weight Loss During Carbohydrate Restriction

Ten healthy volunteers were enrolled and all of them completed the study. The first participants were included on September 18, 2023, and the last examination took place on November 17, 2023. No adverse events were reported and all datasets (*n* = 10) were analysed. During the 14-day study phase, all participants activated their lipolysis and lost 3.0 ± 1.9 kg of body weight (*p* = 0.002). Accordingly, further parameters of body composition, such as BMI, fat mass, and waist and hip circumference also showed significant reductions. Fasting insulin levels decreased by 2.5 ± 1.9 µU/mL (*p* = 0.006; Table 1).

### 3.2. U-Shaped Ketosis Profile in Non-Diabetic Adults

**Figure 1:**
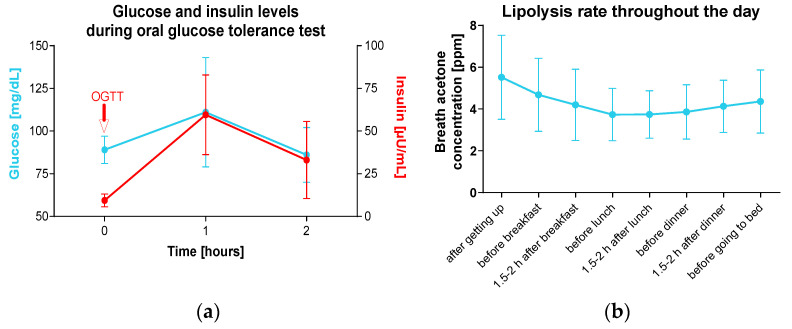
Glucose and insulin levels during oral glucose tolerance test and lipolysis rate throughout the day. Shown are means and standard deviations. (**a**) Before the start of the study (day 1), a standardized oral 75 g glucose tolerance test (OGTT) was performed in the morning on healthy individuals (*n* = 10) without type 2 diabetes. Glucose and insulin concentrations were determined from venous blood at time points 0, 1, and 2 h. (**b**) The participants determined their lipolysis rate for 14 days. For this purpose, the acetone concentration in the breath was measured at eight defined time points during the day using a ketone meter.

Fasting glucose levels of all participants were in the normal, non-diabetic range (89 ± 8 mg/dL), increased to 111 ± 32 mg/dL one hour after the 75 g glucose load, and returned to the normal range after two hours (86 ± 16 mg/dL). Insulin levels increased from fasting 9.4 ± 3.8 µU/mL to 59.5 ± 23.4 µU/mL after one hour and decreased to 33.5 ± 22.6 µU/mL after two hours (Figure 1a). The highest concentration of breath acetone was observed in the morning in a fasted state. The ketosis rate shows a U-shaped daily profile (Figure 1b).

### 3.3. A Single Carbohydrate Meal Disturbed Lipolysis for Several Days

**Figure 2:**
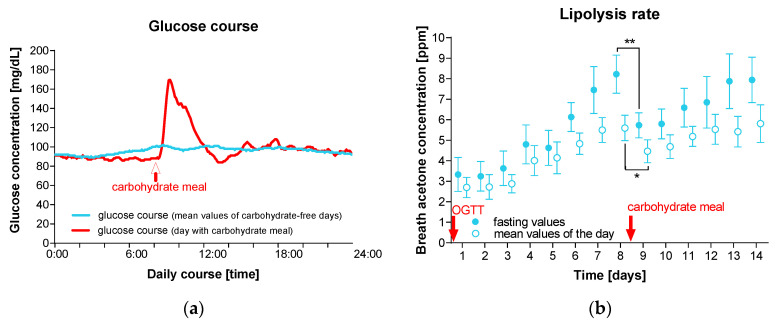
Glucose levels and fat burning rate. The study participants (*n* = 10) followed a ketogenic diet for 14 days. On day 8, a breakfast with digestible carbohydrates (=two white bread rolls with jam) was consumed. (**a**) Glucose concentration was measured over 14 days using continuous glucose monitoring. The graph shows the glucose concentration of the participants throughout the day on the 13 nearly carbohydrate-free days (blue curve) and on the day of carbohydrate consumption (red curve). (**b**) The fat burning rate was determined based on breath acetone concentration. Shown are the mean values and standard errors of the fasting and daily average values. Differences between breath acetone concentrations on day 8 and 9 were calculated using paired *t*-tests (*, *p* < 0.5; **, *p* < 0.01).

Continuous glucose monitoring shows that on days with carbohydrate restriction, glucose levels were stable with minimal fluctuations (mean 96 mg/dL; range 89–102 mg/dL). However, on the day of digestible carbohydrate consumption, glucose levels increased significantly, reaching an average of 170 mg/dL (Figure 2a).

After one week of carbohydrate restriction, the participants achieved stable ketosis (defined as fasting breath acetone concentration ≥ 7.0 ppm) with a mean value of 8.2 ppm on day 8. After the ingestion of the carbohydrate meal, the fasting breath acetone concentration significantly dropped to 5.7 ppm (*p* = 0.0014), and it took 5 days to achieve stable ketosis again (i.e., 7.9 ppm on day 13). This effect can also be observed in the daily average values of breath acetone concentration (Figure 2b).

### 3.4. Increased Insulin Levels Decelerate Ketosis

**Figure 3:**
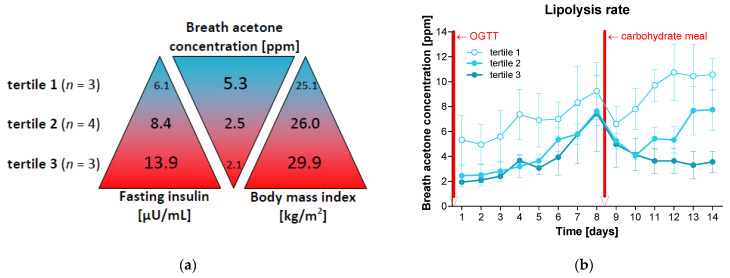
Lipolysis rate depending on fasting insulin. (**a**) The study participants (*n* = 10) were divided into tertiles based on their fasting insulin levels at the beginning of the study, and the respective lipolysis rate (determined based on the average breath acetone concentration over 14 days after waking up) and BMI were compared. Shown are mean values. Thus, tertile 1 contains those persons with the lowest fasting insulin levels, who on the other hand showed the highest breath acetone concentrations and lowest BMI values. In contrast, participants in tertile 3 demonstrated the highest fasting insulin levels, lowest breath acetone concentrations, and highest BMI. (**b**) Shown are means and standard errors of the lipolysis rate over 14 days in the three tertiles.

The stratification of the participants into tertiles based on their fasting insulin levels at baseline (which tended to be negatively associated with baseline breath acetone concentrations and positively with BMI; Figure 3a) showed that individuals with lower fasting insulin levels achieved stable ketosis again after 2 days and persons with medium insulin levels took 5 days, while those with higher baseline values did not reach the stable ketosis range by the end of the study (Figure 3b).

## 4. Discussion

This single-group mechanistic cohort study shows that stable ketosis, leading to activated lipolysis and significant weight reduction, can be achieved after one week of carbohydrate restriction. However, one single carbohydrate meal is sufficient to inhibit ketosis for several days. This effect is most pronounced in individuals with higher fasting insulin levels. Thus, these results deliver a mechanistic insight into lipolysis regulation and underline the negative effects of digestible carbohydrate consumption and insulin action on weight development. We further propose a new technological approach by measuring breath acetone concentration for the self-monitoring of ketosis and non-insulinogenic behaviour. This tool could be used to strengthen motivation and self-empowerment in weight loss approaches. Overweight or obese individuals, especially those with elevated insulin levels, should consistently avoid digestible carbohydrate consumption in order to enable successful lipolysis and weight loss.

As part of the 14-day intervention with the reduction in digestible carbohydrates according to the low-insulin method [23], all participants lost weight. In this context, the magnitude of the reduction in insulin levels is a prognostic factor for weight loss [20]. However, lifestyle intervention studies have unanimously shown that the achieved weight loss becomes diminished and the metabolic control deteriorates as soon as the participants re-adopt old behaviour patterns [29]. It does not have to be a complete return to unhealthy behaviour. Just the re-introduction of meals containing digestible carbohydrates, while maintaining lifestyle changes such as increased physical activity, seems to be sufficient to lead to weight increase [19]. The participants often do not consciously consume sweets or other foods with quickly available carbohydrates, such as white bread. While thinking themselves to be on the safe side, they choose wholemeal bread. Although the glucose increase may be a bit slower, the digestible carbohydrates of breads made from milled whole grain are still split up and lead to insulin release [22,30].

Animal experiments in the 1950s showed that fasting-induced ketosis can be blocked by administering either glucose or insulin. After the injection, blood ketone levels dropped significantly [31]. In fact, insulin or glucose injection inhibits ketosis within 5 min because acetoacetate production in the liver is immediately stopped [32]. In healthy rats, significant resumption in lipogenesis did not occur until at least 3 h after refeeding [32], while in pancreatectomised diabetic rats fatty acid synthesis increased within 30 min after insulin injection [33]. This is in line with the hypothesis that in healthy individuals with metabolic flexibility [16], ketosis could be interrupted without changes in fatty acid oxidation, while in metabolic-impaired individuals the pendulum always swings in the direction of weight gain.

Therefore, it is very important to include tools in a weight-loss-aiming lifestyle intervention that can be used for self-monitoring. Such approaches will make it obvious for the participant if the self-chosen diet is suitable to activate and support lipolysis. In previous trials, we were able to demonstrate the beneficial effects of the self-monitoring of blood glucose (SMBG) not only on glycaemic control and weight development but also on motivation, personal responsibility, and self-empowerment of type 2 diabetes patients [34]. Today, SMBG and even the continuous monitoring of glucose levels are an integral part of guidelines for the therapy of type 2 diabetes [35] and an important tool for detecting glucose peaks. The self-monitoring of breath acetone (SMBA) as a measurement for ketosis level is a new technical approach, which so far has not been regularly used in lifestyle intervention studies. Creating a ketone daily profile helps people to understand which foods are suitable for losing weight and which are not, e.g., due to containing hidden sugars [36]. This information could help you choose suitable foods on the one hand, and to omit unsuitable ones on the other. Thus, it would have an educational effect. Moreover, it should help to maintain motivation and to avoid frustration, which often occurs if despite efforts and renouncement, no success in weight loss can be observed. Continuous glucose monitoring and SMBA are technical developments that close this supply gap. Our data have shown that the breath acetone concentrations are highest after an overnight fast, rendering the fasting state directly after getting up the most suitable time to control for ketosis. Compared to continuous glucose measurement, a big advantage of ketone measurement is non-invasiveness [37]. Overall, ketosis measurement might have the potential to increase the understanding of physiological processes, to enhance people’s self-responsibility, and to avoid relapses. The stratified analysis also shows that carbohydrate restriction is beneficial, especially for people with increased insulin levels. Starvation experiments have shown that ketone levels increase continuously with prolonged fasting, until a plateau is reached after 17 days [38]. People with lower insulin levels are less affected in their ketosis by carbohydrate-containing meals. In general, metabolically healthy people show greater metabolic flexibility, which allows their metabolism to flexibly switch back and forth between carbohydrates and fats for energy production [39]. Ketones themselves act as signalling molecules [40]; thus, ketogenic diets have been shown to be effective in suppressing appetite [41] as well as for the prophylaxis and treatment of obesity-associated illnesses such as type 2 diabetes mellitus [42]. This information can be used to support persons who want to lose weight with individual or personalised offers.

There are strengths and limitations to be mentioned. The small number of participants and a potential bias caused by recruiting volunteers can be seen as limitations of the generalisability of the results. These may also include a lack of precision and reliability, the risk of random variability, and a limited exploration of heterogeneity. However, the study was intended to increase the understanding of the basic mechanisms of ketosis and, according to the sample size calculation, 10 people were sufficient to detect significant differences. Actually, the effects of carbohydrate consumption on lipolysis were uniform. The breath acetone concentrations of all participants dropped, which in turn argues for the generalisability of the results. In further studies, the analysis should be expanded to larger cohorts, such as people with obesity, the metabolic syndrome, or type 2 diabetes. A big advantage is that the findings have a direct application and relevance and can be used in obesity therapy. The metabolic effects of fasting or a ketogenic diet are comparable [43], although the second is better suited for long-term use. In this context, programs that increase the understanding of disease, patients’ therapy involvement, and motivation can provide real benefits.

## 5. Conclusions

This mechanistic analysis shows that the restriction of digestible carbohydrates and the avoidance of insulinogenic behaviours activates lipolysis and leads to significant weight loss within 14 days. In this context, SMBA can be used as an educational and motivating tool during lifestyle interventions. Since a single carbohydrate meal is enough to inhibit lipolysis for several days, weight loss advice allowing cheat days with the intake of digestible carbohydrates is counterproductive. Nutritional and dietary approaches to obesity and type 2 diabetes should take this into account and point out that the consistent restriction of digestible carbohydrates is necessary for successful weight loss.

## Figures and Tables

**Table 1:** Participant characteristics at the start of the study and after 14 days.

Parameter	Day 1	Day 14	Difference After 14 Days	*p*
Sex (women/men) [*n*]	7/3			
Age [years]	48.4 ± 14.3			
Weight [kg]	79.1 ± 18.8	76.2 ± 17.3	−3.0 ± 1.9	**0.002**
Body mass index [kg/m^2^]	26.9 ± 3.9	25.9 ± 3.4	−1.0 ± 0.6	**0.002**
Waist circumference [cm]	90.4 ± 12.7	86.0 ± 12.0	−4.4 ± 3.3	**0.016**
Hip circumference [cm]	100.4 ± 9.0	98.1 ± 8.4	−2.3 ± 1.0	**0.008**
Fat mass [kg]	28.7 ± 9.4	27.1 ± 9.2	−1.6 ± 1.0	**0.004**
Fat mass [%]	36.1 ± 1.0	35.3 ± 6.8	−0.8 ± 1.1	0.059
Visceral fat [L]	2.2 ± 1.4	1.8 ± 1.4	−0.4 ± 0.5	**0.014**
Fat-free mass [kg]	50.4 ± 12.6	49.0 ± 11.5	−1.4 ± 1.6	**0.027**
Fat-free mass [%]	63.9 ± 6.5	64.7 ± 6.8	0.8 ± 1.1	0.059
Skeletal muscle mass [kg]	23.8 ± 7.2	23.0 ± 6.8	−0.8 ± 0.7	**0.006**
Total water mass [L]	37.4 ± 9.0	36.4 ± 8.3	−1.1 ± 1.3	**0.025**
Resting energy expenditure [kcal/day]	1562 ± 298	1531 ± 279	−31 ± 26	**0.002**
Total energy expenditure [kcal/day]	2689 ± 532	2586 ± 460	−103 ± 111	**0.002**
Fasting insulin [µU/mL]	9.4 ± 3.8	6.9 ± 3.7	−2.5 ± 1.9	**0.006**

Shown are means and standard deviations. Differences between the two time points were calculated using the Wilcoxon signed rank test. Significant differences are highlighted in bold.

## Data Availability

Data are available from the corresponding author on request.
